# HIF-1α 1772 C/T and 1790 G/A Polymorphisms Are Significantly Associated with Higher Cancer Risk: An Updated Meta-Analysis from 34 Case-Control Studies

**DOI:** 10.1371/journal.pone.0080396

**Published:** 2013-11-18

**Authors:** Xi Yang, Hong-Cheng Zhu, Chi Zhang, Qin Qin, Jia Liu, Li-Ping Xu, Lian-Jun Zhao, Qu Zhang, Jing Cai, Jian-Xin Ma, Hong-Yan Cheng, Xin-Chen Sun

**Affiliations:** 1 Department of Radiation Oncology, the First Affiliated Hospital of Nanjing Medical University, Nanjing, China; 2 The Comprehensive Cancer Center, Nanjing Drum Tower Hospital and Clinical College of Nanjing Medical University, Nanjing, China; 3 Department of Radiotherapy, Second People's Hospital of Lian Yungang, Lian Yungang, China; 4 Tumor Institute, Nantong Tumor Hospital, Nantong, China; 5 Department of Synthetic Internal Medicine, the First Affiliated Hospital of Nanjing Medical University, Nanjing, China; Duke Cancer Institute, United States of America

## Abstract

**Background:**

HIF-1 activates various genes in cancer progression and metastasis. HIF-1α 1772 C/T and 1790 G/A polymorphisms are reportedly associated with cancer risk; however, the results are inconclusive.

**Methodology/Principal Findings:**

A meta-analysis of 34 studies that involved 7522 cases and 9847 controls for 1772 C/T and 24 studies that involved 4884 cases and 8154 controls for 1790 G/A was conducted to identify the association of C/T and G/A polymorphisms with cancer risk. Odds ratio (OR) and 95% confidence intervals (95% CI) were used to assess the strength of association.

HIF-1α 1772 C/T and 1790 G/A polymorphisms were associated with higher cancer risk in homozygote comparison (1772C/T: TT vs. CC: OR = 2.45, 95% CI: 1.52, 3.96; *P*_heterogeneity_ = 0.028; 1790G/A: AA vs. GG: OR=4.74, 95% CI: 1.78, 12.6; *P*_heterogeneity_ < 0.01), dominant model (1772C/T: TT/CT vs. CC: OR = 1.27, 95% CI: 1.04, 1.55; *P*_heterogeneity_ < 0.01, 1790G/A: AA/GA vs. GG: OR = 1.65, 95% CI: 1.05, 2.60; *P*_heterogeneity_ < 0.01), T allele versus C allele (T vs. C: OR = 1.42, 95% CI: 1.18, 1.70; *P*_heterogeneity_ < 0.01), and A allele versus G allele (A vs. G: OR = 1.83, 95% CI: 1.13, 2.96; *P*_heterogeneity_ < 0.01). On a subgroup analysis, the 1772 C/T polymorphism was significantly linked to higher risks for breast cancer, lung cancer, prostate cancer, and cervical cancer, whereas the 1790 G/A polymorphism was significantly linked to higher risks for lung cancer and prostate cancer. A significantly increased cancer risk was found in both Asians and Caucasians for 1772C/T polymorphism, whereas a significantly increased cancer risk was found in Caucasians in the heterozygote comparison and recessive model for 1790G/A polymorphism.

**Conclusions:**

HIF-1α 1772 C/T and 1790 G/A polymorphisms are significantly associated with higher cancer risk.

## INTRODUCTION

Cancer, which results from complex interactions between genetic and environmental factors, has become a challenging health problem. An increasing number of studies have been performed in the past few years to assess the relationship between genetic variation and cancer risk [1].

Oxygen (O_2_) concentration in tumor tissues is significantly lower than that in the surrounding normal tissues. Many studies have focused on hypoxia because of its function in maintaining tumor microenvironments [2]. Hypoxic tumor microenvironment initiates multiple cellular responses, such as proliferation and angiogenesis, triggering the development and progression of cancer. In general, hypoxia may regulate tumor cell phenotypes by altering genes that are sensitive to O_2_ pressure [3]. Studies have demonstrated that HIF-1 has an important function in the development and progression of cancer by activating various genes associated with angiogenesis, cell adhesion, erythropoiesis, and glucose transportation [4]. HIF-1 is a heterodimer consisting of an oxygen-sensitive subunit HIF-1α and a constitutively expressed subunit HIF-1β; it is degraded rapidly through the von Hippel–Lindau-mediated ubiquitin–proteasome pathway under normoxia conditions [5]. Recent studies have shown that HIF-1α is overexpressed in many human cancers with advanced tumor grade, suggesting that HIF-1α acts as an independent factor of cancer prognosis [6]. 

The human HIF-1α gene, which is located at chromosome 14q21–24, is composed of 15 exons. It codes for a 3919 bp cDNA and produces an 826 amino acid protein. Single nucleotide polymorphisms (SNPs) in coding regions can mediate amino acid changes and affect the structure and biological activity of the translated protein [7]. The most widely studied HIF-1α polymorphisms are 1772 C/T (Pro582Ser, rs11549465) and 1790 G/A (Ala588Thr, G1790A, rs11549467), which induce proline-to-serine and alanine-to-threonine amino acid substitutions, respectively. Both polymorphic variants can significantly elevate transcriptional activity than the wild type under both hypoxic and normoxic conditions in in vitro studies [8]. Moreover, both polymorphisms are associated with increased tumor microvessel density, thereby contributing to the development and progression of cancer [9].

HIF-1α 1772 C/T and 1790 G/A genetic polymorphisms were previously suggested to be responsible for the risk for various types of cancer. However, the results of epidemiological studies are inconsistent [10–12]. Thus, the relationship between HIF-1α polymorphisms and cancers requires further investigation. Accordingly, we performed a meta-analysis on eligible case–control studies to produce a more powerful estimation of the association of HIF-1α 1772 C/T and 1790 G/A polymorphisms with cancer risk.

## Methods

### Identification and eligibility of relevant studies

All studies published before June 26, 2013 that investigated the association of HIF-1α 1772 C/T and 1790 G/A polymorphisms with cancer risk were considered in this meta-analysis. A systematic search of literature was carried out using PubMed and Embase. The keywords used for the search were “hypoxia-inducible factor-1” or “HIF-1” concatenated with “SNP,” “polymorphism,” “mutation,” or “variant” and “tumor,” “cancer,” “carcinoma,” or “malignancy.” Only studies with complete data on the comparison of HIF-1α 1772 C/T or 1790 G/A polymorphisms between cancer patients and controls were selected. Case reports, animal studies, review articles, editorials, abstracts, reports with incomplete data, and studies based on pedigree data were excluded.

### Data extraction

Two investigators (Yang and Zhu) independently reviewed the articles to exclude irrelevant and overlapping studies. The results were compared, and disagreements were resolved by discussion and consensus. We only included the publication that reported the most extensive information when overlapping articles were found. The following data were extracted for each study: first author, year of publication, country, ethnicity, control source, cancer type, Hardy–Weinberg equilibrium, and the number of cases and controls for each genotype.

### Statistical analysis

STATA (version 11.0; StataCorp, College Station, Texas, USA) was used for the meta-analysis. All genotype models for the two HIF-1α polymorphisms were evaluated. We also conducted subgroup analyses by cancer type, ethnicity, and source of control. For cancer type subgroups, we included the subgroup that contained more than three studies.

The existence of heterogeneity between studies was ascertained by Q-statistic. The pooled odds ratio (OR) was estimated with models based on fixed-effects or random-effects assumptions. A random-effects model was used when the significant Q statistic (*P* < 0.1) indicated the presence of heterogeneity in the studies. Otherwise, a fixed-effects model was selected. The 95% confidence interval (CI) of OR was also calculated. The distribution of genotypes in the controls was checked for Hardy–Weinberg equilibrium. Studies with controls not in the Hardy–Weinberg equilibrium were subjected to sensitivity analysis.

The publication bias among the studies was assayed. Funnel plots of the HIF-1α 1772 C/T polymorphism for T allele versus C allele and the HIF-1α 1790 G/A polymorphism for A allele versus G allele were built to search for any evidence of publication bias. An assymetric funnel plot is indicative of publication bias, whereas a symmetric funnel plot implies the absence of publication bias. Egger’s test, estimated by MIX 1.7 software (Kitasato Clinical Research Center, Kitasato University, Japan), was performed to measure funnel plot asymmetry.

## Results

### Characteristics of eligible studies

**Figure 1:**
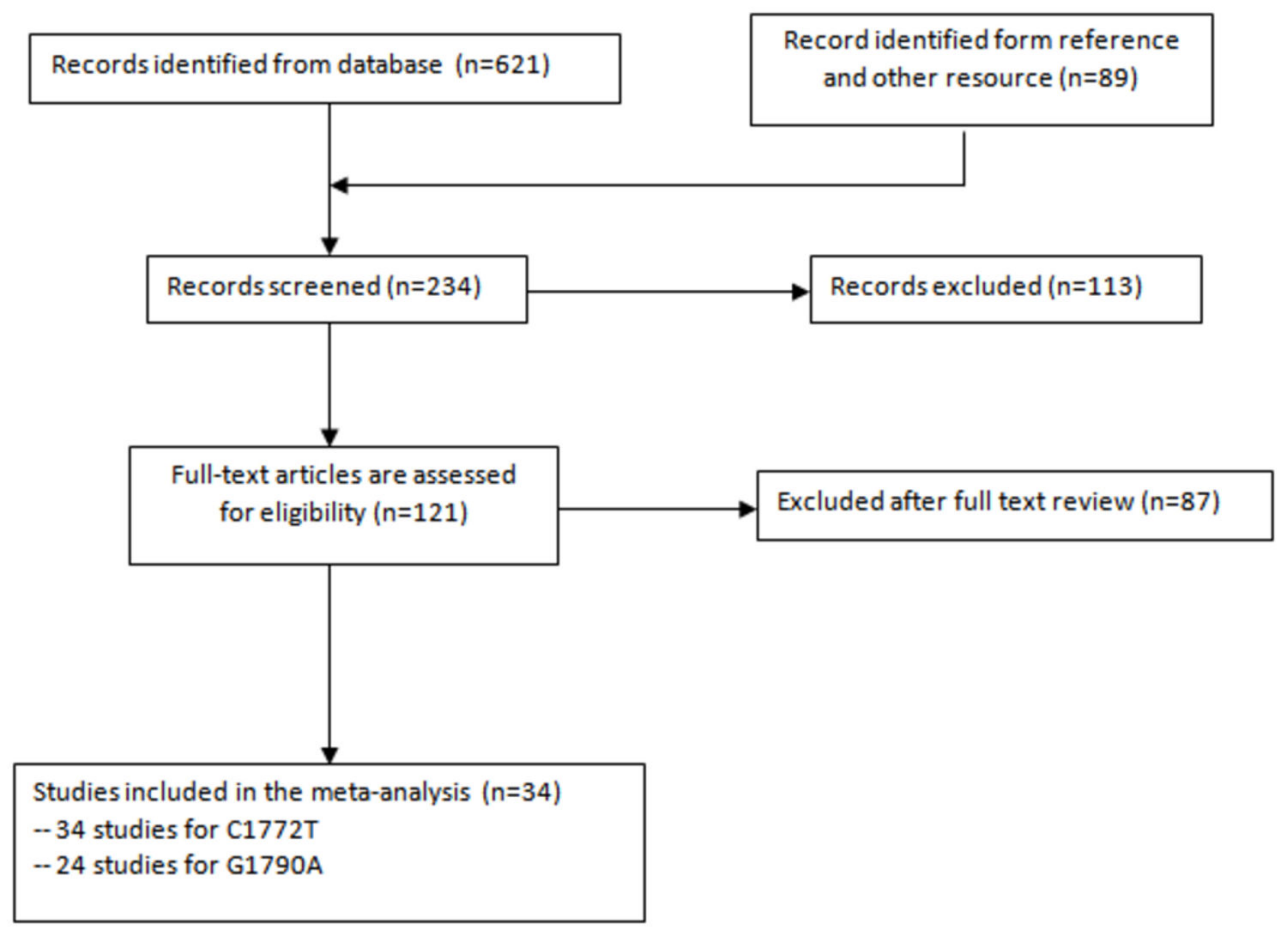
Reference search and selection of studies in the meta-analysis.

The flow diagram illustrates the main reasons for study exclusion (Figure 1). The selected study characteristics are summarized in Tables 1 and 2. Thirty-four relevant case–control studies concerning the 1772 C/T and 1790 G/A polymorphisms and cancer risk were included in the meta-analysis. Of the 34 studies, 5 concentrated on prostate cancer [13–17], 5 on breast cancer [18–22], 4 on colorectal cancer [23–26], 3 on oral cancer [27–29], 3 on lung cancer [30–32], 2 on pancreatic cancer [33,34], 2 on renal cell carcinoma [35,36], 2 on cervical cancer [37,38], 1 on ovarian cancer, endometrial cancer, and cervical cancer [39], and 7 concentrated separately on esophageal squamous cell carcinoma [40], endometrial cancer [9], liver cancer [41], gastric cancer [42], glioma [43], bladder cancer [44], and head and neck squamous cell carcinoma [8]. Among the eligible studies, 34 presented data on 1772 C/T polymorphism and 24 presented data on 1790 G/A polymorphism. For the 1772 C/T polymorphism, the distribution of the genotypes in the control groups in 5 studies was not in Hardy–Weinberg equilibrium [9,13,28,35,36]. For the 1790 G/A polymorphism, the distribution of the genotypes in the control groups in one study was not in Hardy–Weinberg equilibrium [36]. Among the eligible studies, 1 study provided data on three types of cancer (endometrial cancer, ovarian cancer, and cervical cancer) for both polymorphisms [39].

**Table 1:** Characteristics of eligible studies for the association between the 1772 C/T polymorphism and cancer risk.

First Author (Reference)	Year	Country	Ethnicity	Control Source	Cancer Type	Cases			Controls			HWE
						CC	CT	TT	CC	CT	TT	
Tanimoto	2003	Japan	Asian	PB	Head and neck squamous cell carcinoma	45	10	0	98	12	0	0.545
Kuwai	2004	Japan	Asian	PB	Colorectal cancer	100	0	0	89	11	0	0.561
Ling	2005	China	Asian	PB	Esophogeal sqaumous cell carcinoma	84	11	0	93	11	0	0.569
Kim	2008	Korea	Asian	HB	Breast cancer	81	8	1	93	9	0	0.641
Lee	2008	Korea	Asian	PB	Breast cancer	1207	119	6	1245	123	1	0.25
Nadaoka	2008	Japan	Asian	PB	Transitional cell carcinoma of bladder	197	22		419	42		0.35
Chen	2009	China	Asian	PB	Oral cancer	163	10	1	334	13	0	0.722
Li	2009	China	Asian	PB	Gastric cancer	83	4	0	93	13	0	0.501
Naidu	2009	Malaysia	Asian	PB	Breast cancer	294	100	16	222	50	3	0.922
Chai	2010	China	Asian	HB	Cervical cancer	65	25	7	94	21	2	0.52
Hsiao	2010	China	Asian	HB	Hepatocellular carcinoma	94	8	0	334	13	0	0.722
Kang	2011	Korea	Asian	PB	Colorectal cancer	38	12		46	4		
Kim	2011	Korea	Asian	HB	Cervical cancer	177	22	0	187	27	0	0.325
Putra	2011	Japan	Asian	HB	Lung cancer	74	9	0	98	12	0	0.545
Wang	2011	China	Asian	HB	Pancreatic cancer	209, (198)	54	0	242	29	0	0.352
Xu	2011	China	Asian	HB	Glioma	121	27	2	135	14	1	0.354
Li	2012	China	Asian	HB	Prostate cancer	612	48	2	659	57	0	0.267
Clifford	2001	UK	Caucasian	PB	Renal cell carcinoma	30	5	0	110	27	6	0.018
Ollerenshaw	2004	UK	Caucasian	PB	Renal cell carcinoma	16	54	90	1	90	71	<0.001,
Fransen	2006	Sweden	Caucasian	PB	Colorectal cancer	167	28	3	213	43	2	0.916
Konac	2007	Turkey	Caucasian	HB	Endometrial, ovarian, and cervical cancer	48	40	14	68	37	2	0.229
Orr-Urtreger	2007	Israel	Caucasian	PB	Prostate cancer	287	99	16	217	80	3	0.137
Horre´e	2008	Netherlands	Caucasian	PB	Endometrial cancer	50	5	3	463	84	12	0.001
Apaydin	2008	Turkey	Caucasian	PB	Breast cancer	79	21	2	68	29	5	0.415
Foley	2009	Ireland	Caucasian	PB	Prostate cancer	65	30	0	175	13	0	0.623
Muñoz-Guerra	2009	Spain	Caucasian	PB	Oral cancer	57,	6	7	113	27	8	0.001
Konac	2009	Turkey	Caucasian	HB	Lung cancer	110,	31	0	111	43	2	0.335
Knechtel	2010	Austrila	Caucasian	HB	Colorectal cancer	291	77		1773	383		>0.05
Ruiz-Tovar	2012	Spian	Caucasian	PB	Pancreatic cancer	47	1	11	116	28	8	0.002
Kuo	2012	China	Caucasian	HB	Lung cancer	153	94	38	216	73	11	0.132
Alves	2012	Brazil	Caucasian	PB	Oral cancer	0	1	39	0	85	3	<0.001
Zagouri	2012	Greece	Caucasian	HB	Breast cancer	98	15	0	107	17	0	0.413
Chau	2005	USA	Mixed	PB	Prostate cancer	161	29	6	179	14	3	<0.001
Li	2007	USA	Mixed	PB	Prostate cancer	818	209	14	175	13	0	0.623

**Table 2:** Characteristics of eligible studies for the association between the 1790 A/G polymorphism and cancer risk.

First Author (Reference)	Year	Country	Ethnicity	Control Source	Cancer Type	Cases			Controls			HWE
						GG	GA	AA	GG	GA	AA	
Tanimoto	2003	Japan	Asian	PB	Head and neck squamous cell carcinoma	51	4	0	101	9	0	0.655
Kim	2008	Korea	Asian	HB	Breast cancer	87	3	0	94	7	1	0.06
Nadaoka	2008	Japan	Asian	PB	Transitional cell carcinoma of bladder	204	15		421	40		0.25
Chen	2009	China	Asian	PB	Oral cancer	333	14	0	153	20	1	0.697
Li	2009	China	Asian	PB	Gastric cancer	74	13	0	100	6	0	0.764
Naidu	2009	Malaysia	Asian	PB	Breast cancer	332	72	6	232	41	2	0.898
Hsiao	2010	China	Asian	HB	Hepatocellular carcinoma	27	8	0	200	7	0	0.805
Kim	2011	Korea	Asian	HB	Cervical cancer	187	12	0	200	13	1	0.136
Putra	2011	Japan	Asian	HB	Lung cancer	72	9	2	101	9	0	0.655
Wang	2011	China	Asian	HB	Pancreatic cancer	198	64	1	249	22	0	0.486
Li	2012	China	Asian	HB	Prostate cancer	614	47	1	685	31	0	0.554
Clifford	2001	UK	Caucasian	PB	Renal cell carcinoma	35	0	0	140	4	0	0.866
Ollerenshaw	2004	UK	Caucasian	PB	Renal cell carcinoma	65	67	14	239	39	10	<0.001
Fransen	2006	Sweden	Caucasian	PB	Colorectal cancer	189	89	0	247	9	0	0.775
Konac	2007	Turkey	Caucasian	HB	Endometrial, ovarian, and cervical cancer	100	2	0	107	0	0	1
Orr-Urtreger	2007	Israel	Caucasian	PB	Prostate cancer	198	2	0	298	2	0	0.954
Apaydin	2008	Turkey	Caucasian	PB	Breast cancer	102	0	0	94	4	0	0.837
Muñoz-Guerra	2009	Spain	Caucasian	PB	Oral cancer	40	21	3	130	9	0	0.693
Konac	2009	Turkey	Caucasian	HB	Lung cancer	140	1	0	152	2	0	0.936
Knechtel	2010	Austrila	Caucasian	HB	Colorectal cancer	356	11		2080	76		>0.05
Ruiz-Tovar	2012	Spian	Caucasian	PB	Pancreatic cancer	54	2	3	142	10	0	0.675
Kuo	2012	China	Caucasian	HB	Lung cancer	150	1	41	215	74	11	0.154
Alves	2012	Brazil	Caucasian	PB	Oral cancer	2	1	37	81	7	0	0.698
Li	2007	USA	Mixed	PB	Prostate cancer	1053	13	0	1247	17	0	0.81

### Summary statistics

The meta-analysis for the HIF-1α 1772 C/T polymorphism included 7522 cases and 9847 controls. The prevalence of the CC genotype was the highest, allele C was the most frequent, and the prevalence of the TT genotype was the lowest in both case and control groups. 

The meta-analysis for the HIF-1α 1790 G/A polymorphism included 4884 cancer cases and 8154 controls. The prevalence of the GG genotype was the highest, allele G was the most frequent, and the prevalence of the AA genotype was the lowest in both case and control groups.

### Overall analysis

**Figure 2:**
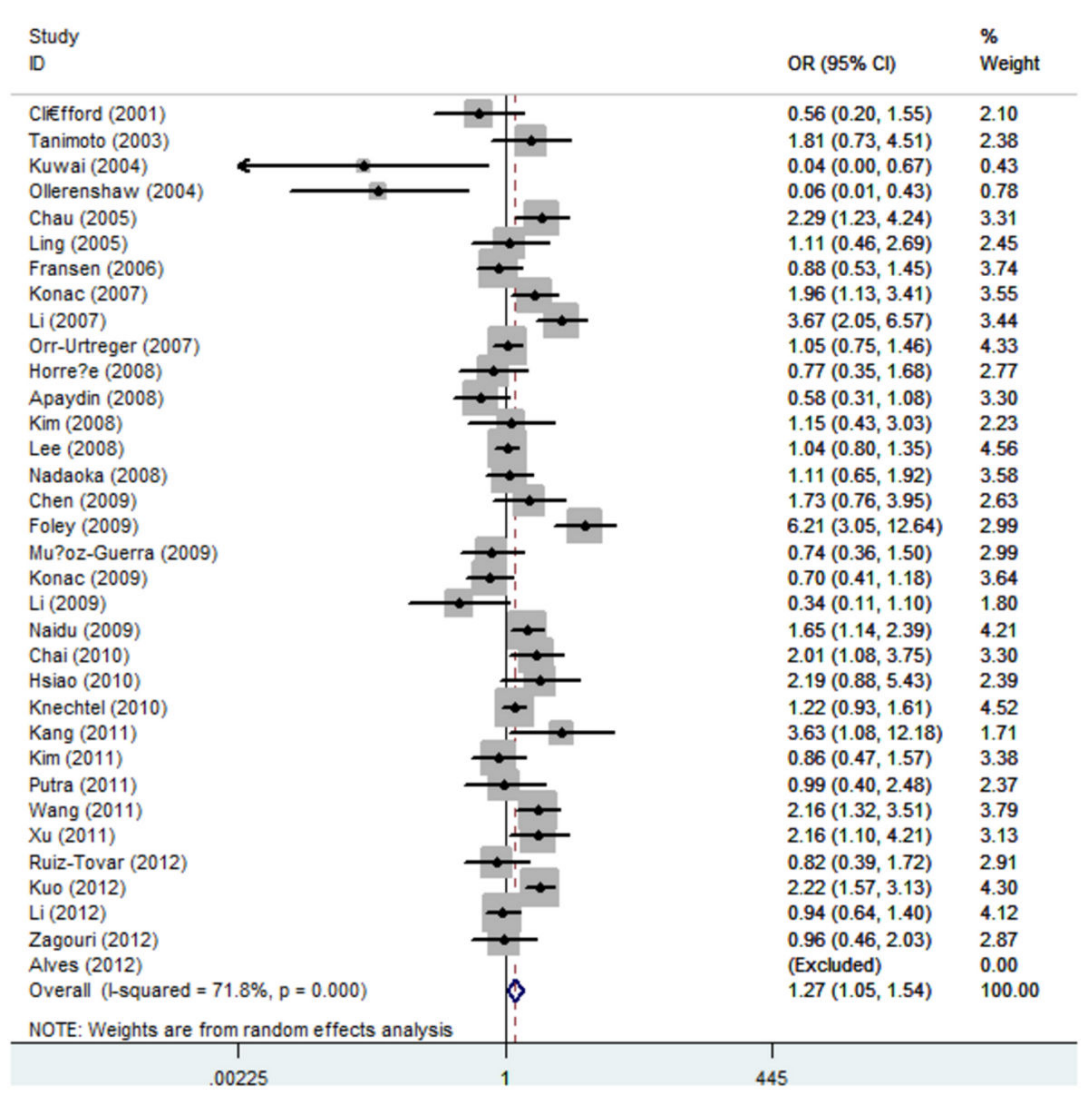
Forest plot of dominant model for overall comparison (1772 C/T, **TT**/**CT** vs. CC).

**Figure 3:**
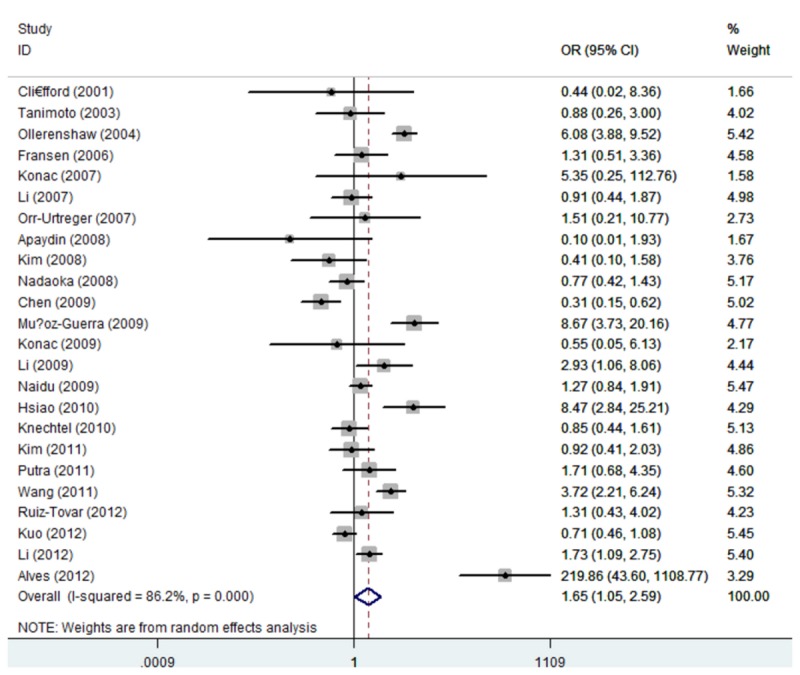
Forest plot of dominant model for overall comparison (1790 G/A, **AA**/**GA** vs. GG).

**Figure 4:**
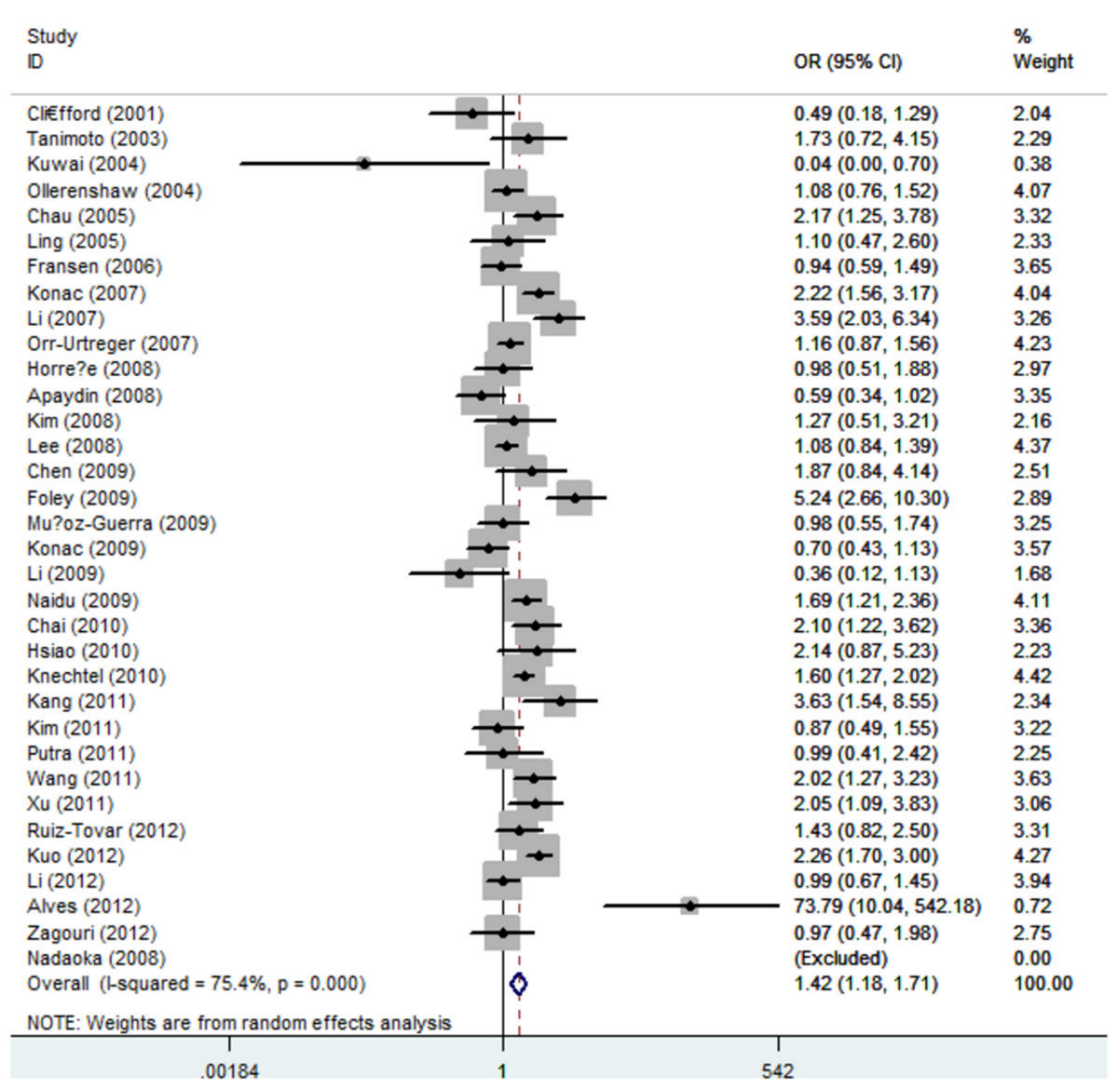
Forest plot of overall comparison (1772 C/T, T allele vs. C allele).

**Figure 5:**
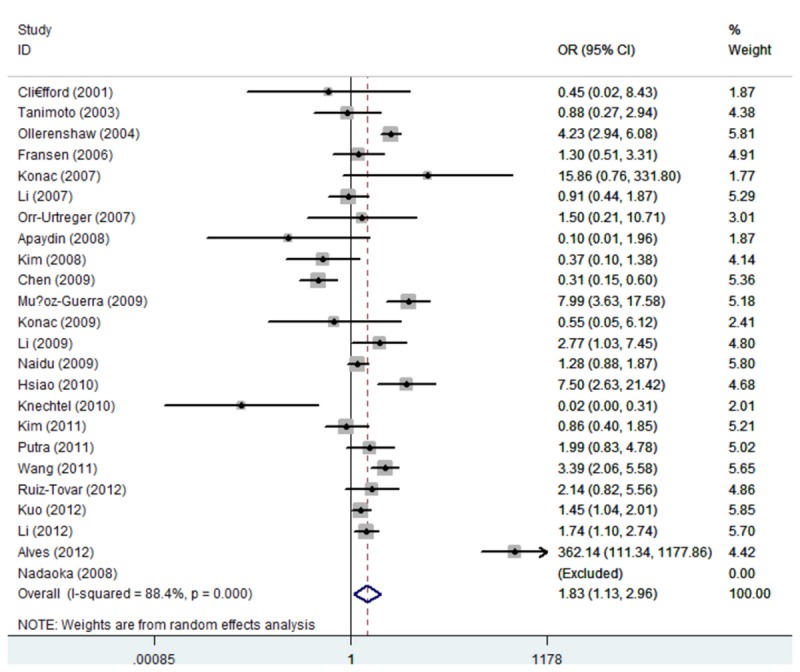
Forest plot of overall comparison (1790 G/A, A allele vs. G allele).

Upon pooling of all eligible studies, we observed that both 1772 C/T and 1790 G/A polymorphisms were significantly associated with cancer risk in homozygote comparison (1772C/T: TT vs. CC: OR=2.45, 95% CI: 1.52, 3.96; *P*_heterogeneity_ = 0.028; 1790G/A: AA vs. GG: OR = 4.74, 95% CI: 1.78, 12.6; *P*_heterogeneity_ < 0.01), dominant model (1772C/T: TT/CT vs. CC: OR=1.27, 95% CI: 1.04, 1.55; *P*_heterogeneity_ < 0.01, 1790G/A: AA/GA vs. GG: OR = 1.65, 95% CI: 1.05, 2.60; *P*_heterogeneity_ < 0.01) (Figures 2 and 3), recessive model (1772C/T: TT vs. CC/CT: OR = 3.18, 95% CI: 1.92, 5.29; *P*_heterogeneity_ < 0.01, 1790G/A: AA vs. GG/GA: OR = 4.39, 95% CI: 1.61,11.9; *P*_heterogeneity_ < 0.01), T allele versus C allele (T vs. C: OR = 1.42, 95% CI: 1.18, 1.70; *P*_heterogeneity_ < 0.01), and A allele versus G allele (A vs. G: OR = 1.83, 95% CI: 1.13,2.96; *P*_heterogeneity_ < 0.01) (Figures 4 and 5). The association strength between HIF-1α polymorphism and cancer risk is shown in Table 3. No significant association was found in heterozygote comparison (1772C/T: CT vs. CC: OR = 1.15, 95% CI: 0.92, 1.45; *P*_heterogeneity_ < 0.01, 1790G/A: GA vs. GG: OR = 1.35, 95% CI: 0.82, 2.21; *P*_heterogeneity_ < 0.01). However, the 1772C/T polymorphism was significantly associated with cancer in the heterozygote model (CT vs. CC: OR = 1.29, 95% CI: 1.04, 1.62; P_heterogeneity_ < 0.01) when studies not in the Hardy–Weinberg equilibrium were excluded. 

**Table 3:** Main results of the meta-analysis for the association of HIF1A gene 1772 C/T and 1790 G/A polymorphisms with cancer risk.

1772 C/T polymorphisms (rs11549465)			TT VS CC		CT VS CC		TT/CT VS CC		TT VS CT/CC		T allele VS C allele	
	N	Sample size	OR^a^	*P* ^b^	OR^a^	*P* ^b^	OR^a^	*P* ^b^	OR^a^	*P* ^b^	OR^a^	*P* ^b^
Total	34	7522	**2.45 (1.52–3.96)**	0.028	1.15 (0.92–1.45)	<0.001	**1.27 (1.05–1.55)**	<0.001	**3.18 (1.92–5.29)**	<0.001	**1.42 (1.18–1.70)**	<0.001
Total in HWE	25	6575	**3.65 (2.47–5.40)**	0.318	**1.29 (1.04–1.62)**	<0.001	**1.35 (1.10–1.65)**	<0.001	**3.38 (2.29–5.00)**	0.476	**1.40 (1.15–1.71)**	<0.001
Cancer types												
Breast cancer	5	2047	**2.30 (1.08–4.91)**	0.084	1.07 (0.88–1.29)	0.188	1.12 (0.92–1.35)	0.711	**2.27 (1.06–4.87)**	0.120	1.09 (0.76–1.55)	0.022
Lung cancer	3	509	1.41 (0.07–30.4)	0.044	1.13 (0.59–2.19)	0.018	**1.50 (1.15–1.96)**	0.688	**3.27 (1.73–6.17)**	0.065	1.19 (0.50–2.86)	<0.001
Oral cancer	3	284	2.01 (0.75–5.41)	0.463	0.85 (0.24–2.97)	0.047	1.04 (0.61–1.78)	0.823	22.8 (0.28–1888)	<0.001	3.93 (0.61–25.4)	<0.001
Colorectal cancer	4	627	1.91 (0.32–11.6)		0.24 (0.01–5.51)	0.027	1.10 (0.87–1.38)	0.744	1.97 (0.33–11.9)		1.36 (0.68–2.70)	0.002
Prostate cancer	5	2396	**3.68 (1.58–8.55)**	0.871	**2.02 (1.01–4.07)**	<0.001	**2.10 (1.08–4.09)**	0.028	**3.52 (1.52–8.16)**	0.847	**2.06 (1.15–3.68)**	<0.001
Cervical cancer	3	328	**10.1 (3.12–32.6)**	0.153	1.37 (0.92–2.02)	0.099	**1.63 (1.12–2.37)**	0.158	**8.26 (2.64–25.9)**	0.236	1.89 (0.84–4.26)	0.002
Others	13	1331	1.68 (0.42–6.80)	<0.001	0.97 (0.56–1.68)	<0.001	1.20 (0.98–1.47)	0.512	**1.99 (1.40–2.84)**	0.100	1.37 (0.96–1.97)	<0.001
Ethnicities												
Caucasian	15	2151	1.70 (0.81–3.55)	0.001	0.86 (0.57–1.31)	<0.001	1.05 (0.76–1.46)	<0.001	**2.97 (1.44–6.14)**	<0.001	1.32 (0.99–1.75)	<0.001
Asian	17	4134	**4.42 (2.07–9.43)**	0.997	1.25 (0.98–1.60)	0.010	**1.33 (1.06–1.68)**	0.006	**4.12 (1.93–8.77)**	0.955	**1.40 (1.11–1.78)**	**0.002**
Mixed	2	1237	3.13 (0.90–10.8)	0.500	**2.98 (1.92–4.63)**	0.372	**3.05 (2.00–4.66)**	0.269	2.77 (0.80–9.54)	0.646	**2.91 (1.96–4.32)**	**0.208**
Source of control												
PB	21	4944	**1.92 (1.05–3.50)**	0.037	0.99 (0.69–1.41)	<0.001	1.17 (0.87–1.57)	<0.001	**3.14 (1.60–6.16)**	<0.001	**1.40 (1.06–1.84)**	**<0.001**
HB	13	2578	**4.38 (2.64–7.47)**	0.486	**1.32 (1.13–1.57)**	0.023	**1.39 (1.09–1.77)**	0.002	**3.88 (2.32–6.51)**	0.569	**1.46 (1.16–1.85)**	**<0.001**
1790 G/A polymorphisms (rs11549465)			AA VS GG		GA VS GG		AA/GA VS GG		AA VS GA/GG		A allele VS G allele	
	N	Sample size	OR^a^	*P* ^b^	OR^a^	*P* ^b^	OR^a^	*P* ^b^	OR^a^	*P* ^b^	OR^a^	*P* ^b^
Total	24	5136	**4.74 (1.78–12.6)**	0.002	1.35 (0.82–2.21)	<0.001	**1.65 (1.05–2.60)**	<0.001	**4.39 (1.61–11.9)**	0.001	**1.83 (1.13–2.96)**	**<0.001**
Total in HWE	23	5090	**4.68 (1.34–16.3)**	0.001	1.23 (0.77–1.98)	<0.001	1.53 (0.99–2.36)	<0.001	**4.65 (1.35–16.0)**	0.001	**1.83 (1.13–2.96)**	<0.001
Cancer types												
Breast cancer	3	521	1.44 (0.38–5.44)	0.336	1.03 (0.70–1.52)	0.115	1.05 (0.72–1.53)	0.077	1.41 (0.37-5.37)	0.356	1.07 (0.75-1.52)	0.055
Lung cancer	3	362	5.42 (2.75–10.7)	0.866	0.26 (0.01–7.10)	<0.001	0.82 (0.56–1.19)	0.226	**7.11 (3.61–14.0)**	0.975	**1.48 (1.09-2.00)**	0.575
Oral cancer	3	375	20.7(0.10–4519)	<0.001	2.21 (0.18–26.9)	<0.001	7.81 (0.27–224)	<0.001	17.5 (0.10–3257)	<0.001	9.34 (0.23-388)	<0.001
Prostate cancer	3	1865	3.35 (0.14–82.3)		1.41 (0.97–2.07)	0.365	1.44 (0.98–2.10)	0.340	3.25 (0.13–79.9)		**1.45 (1.00-2.11)**	0.330
Others	14	1542	**4.81 (2.34–9.87)**	0.460	1.70 (0.99–2.90)	<0.001	1.80 (0.99–3.26)	<0.001	**3.01 (1.47–6.21)**	0.367	**1.91 (1.01-3.58)**	<0.001
Ethnicities												
Caucasian	12	1635	**17.4 (4.01-75.3)**	0.001	1.09 (0.33–3.58)	<0.001	2.19 (0.90–5.34)	<0.001	**15.8(3.42–72.9)**	<0.001	2.27 (0.92-5.58)	<0.001
Asian	11	2435	1.44 (0.60-3.46)	0.522	1.45 (0.85–2.46)	<0.001	1.36 (0.83–2.24)	<0.001	1.41 (0.58–3.39)	0.508	1.42 (0.84-2.40)	<0.001
Source of control												
PB	14	3013	**9.69 (1.41-66.7)**	<0.001	1.40 (0.71–2.74)	<0.001	1.80 (0.89–3.64)	<0.001	**8.08 (1.12–58.1)**	<0.001	2.10 (0.95-4.68)	<0.001
HB	10	2123	**4.08 (2.26-7.37)**	0.401	1.23 (0.53–2.86)	<0.001	1.47 (0.85–2.55)	<0.001	**5.02 (2.79–9.02)**	0.278	1.50 (0.86-2.62)	<0.001

^a^ Random-effects model was used when the *P* value for the heterogeneity test was < 0.05; otherwise, fixed-effects model was used.

^b^ *P* Value of Q-Test for the Heterogeneity Test

N: number of studies included; OR: odds ratio; PB: population-based; HB: hospital-based; HWE= Hardy–Weinberg equilibrium.

One study contained detailed data on ovarian cancer, endometrial cancer, and cervical cancer. We used the combined data for the overall analysis and the separate data for the subgroup analysis by cancer type.

### Subgroup analyses

Subgroup analyses were performed to investigate the effect of cancer type, ethnicity, and source of control. For cancer type, the 1772C/T polymorphism demonstrated an increased risk for breast cancer, lung cancer, prostate cancer, cervical cancer, and other cancers in various models. In the subgroup analyses of “oral cancer” and “colorectal cancer,” we did not find any significant association between the 1772C/T polymorphism and cancer risk. The 1790G/A polymorphism exhibited an increased cancer risk for lung cancer in the homozygote and recessive models (AA vs. GG: OR = 5.42, 95% CI: 2.75, 10.7; *P*_heterogeneity_ = 0.866; AA vs. GG/GA: OR = 7.11, 95% CI: 3.61, 14.0; *P*_heterogeneity_ = 0.975; A vs. G: OR = 1.48, 95% CI: 1.09, 2.00; *P*_heterogeneity_ = 0.575) and for prostate cancer (A vs. G: OR = 1.45, 95% CI: 1.00, 2.11; *P*_heterogeneity_ = 0.330). We found a significant association between 1772C/T and 1790G/A polymorphisms and cancer risk in both population-based and hospital-based studies.

However, ethnicity significantly affected cancer susceptibility. For the 1772C/T polymorphism, a significantly increased cancer risk was found in both Asians and Caucasians. For the 1790G/A polymorphism, a significantly increased cancer risk was found in Caucasians in the heterozygote comparison (AA vs. GG: OR = 17.4, 95% CI: 4.01, 75.3; *P*_heterogeneity_ < 0.01) and recessive model (GG/GA: OR = 15.8, 95% CI: 3.42, 72.9; *P*_heterogeneity_ < 0.01). However, no significant association between these polymorphisms and cancer risk was found in Asians. These results revealed that the effect of HIF-1α polymorphisms on cancer was associated with ethnicity. 

### Sensitivity analysis

Sensitivity analysis was performed to explore the influence of an individual study on the pooled results by deleting a single study each time from the pooled analysis. The results showed that no individual study significantly affected the pooled OR because no substantial change was found (figure not shown).

### Publication bias

**Figure 6:**
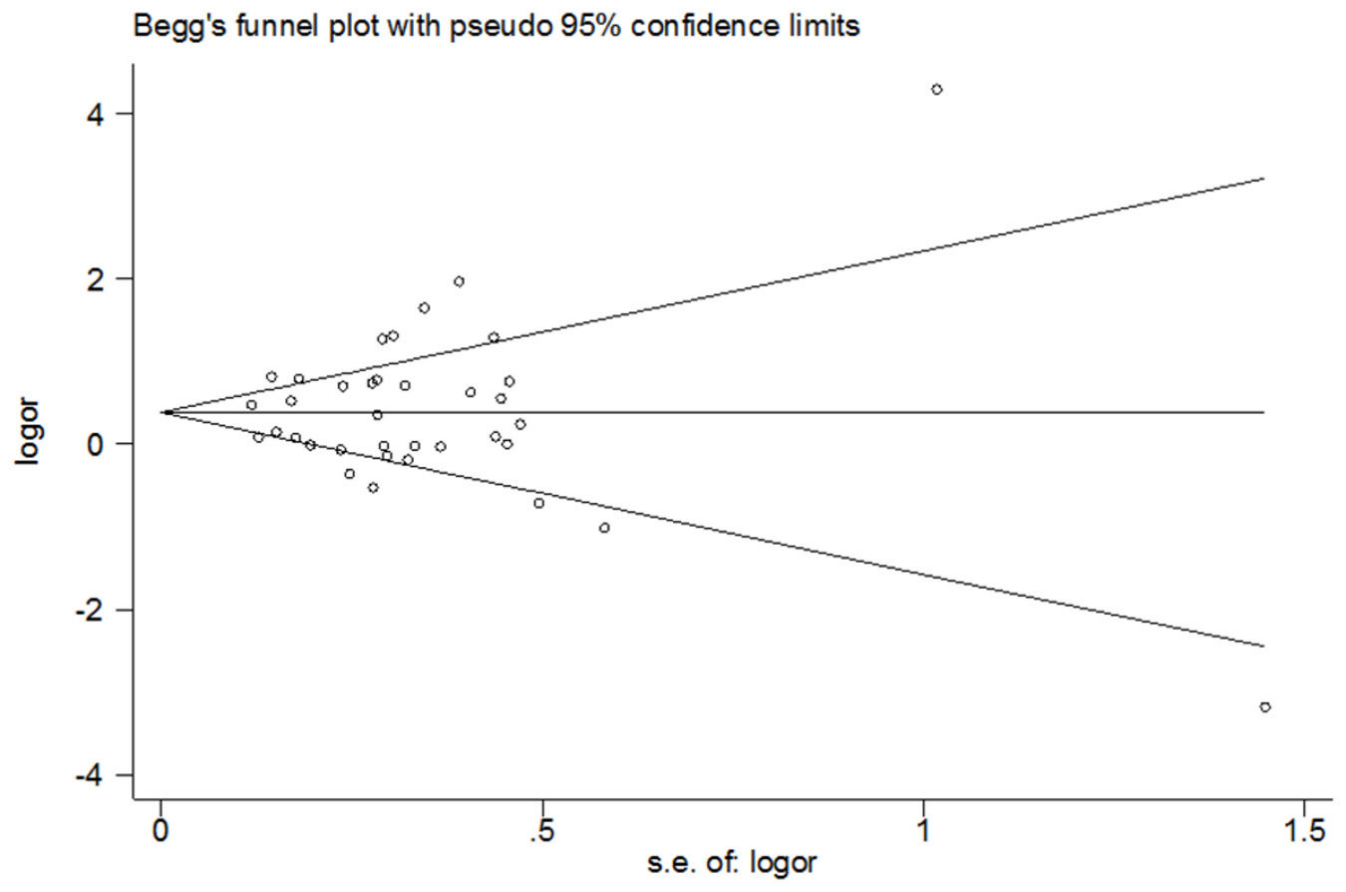
Funnel plot of heterozygote comparison (1772 C/T, T allele vs. C allele).

**Figure 7:**
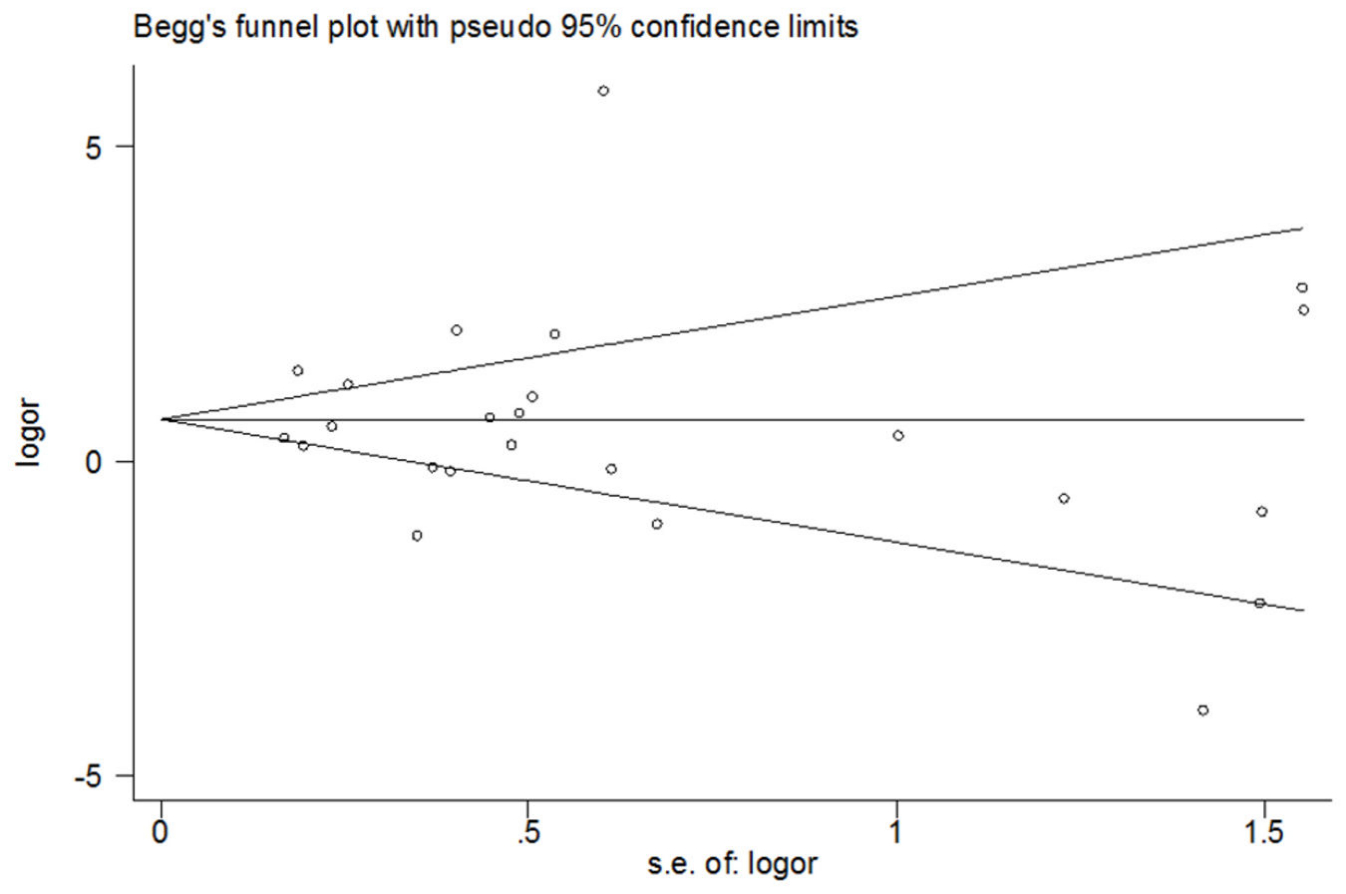
Funnel plot of heterozygote comparison (1790 G/A, A allele vs. G allele).

Publication bias was assessed by Begg’s funnel plot and Egger’s test. Begg’s funnel plot for the 1772 C/T polymorphism is shown in Figure 6 (*P* = 0.589 for T allele vs. C allele). Egger’s test was performed for statistical analysis, and no publication bias was detected (*P* =0.481 for T allele vs. C allele). The results of Begg’s and Egger’s tests for the 1790 G/A polymorphism were *P* = 0.785 and *P* = 0.870, respectively, for A allele versus G allele (Figure 7). Overall, no publication bias was detected in the data.

## Discussion

HIF-1 has an important function in cancer progression and metastasis by activating various genes that are linked to the regulation of angiogenesis, cell survival, and energy metabolism [3]. The presence of T and A variant alleles of HIF-1α 1772, namely, C/T and 1790 G/A polymorphisms, are associated with high transcriptional abilities and protein synthesis in vitro [8]. In vivo studies related these genetic variations to many aggressive clinical features of cancer, such as the ulcerative growth pattern in colorectal tumors, suggesting that HIF-1α polymorphism is associated with cancer [45]. However, studies on the association of HIF-1α 1772 C/T and 1790 G/A polymorphisms with cancer are conflicting. In 2009, Zhao [10] conducted a meta-analysis using 16 case–control studies and concluded that 1772 C/T is significantly associated with higher cancer risk and that 1790 G/A is only significantly associated with breast cancer. Liu [12] performed a similar meta-analysis from 22 case–control studies, including 5552 cases and 8044 controls for 1772 C/T and 3381 cases and 5830 controls for 1790 G/A, and one study evaluated cancer prognosis by polymorphism [45]. This previous study concluded that the 1790 G/A polymorphism and not the 1772 C/T polymorphism is significantly associated with cancer risk. In the present study, we performed an updated meta-analysis from 34 case–control studies that involved 7522 cases and 9847 controls for 1772 C/T polymorphism and 4884 cases and 8154 controls for 1790 G/A polymorphism. 

 In the present meta-analysis, we investigated the association of HIF-1α 1772 C/T and 1790 G/A polymorphisms with cancer risk. Subgroup analyses by cancer type and ethnicity were also performed. Our analyses showed that both 1772C/T and 1790G/A polymorphisms were significantly associated with cancer risk. In the subgroup study, various types of cancers, such as breast cancer, lung cancer, prostate cancer, and cervical cancer, were associated with 1772C/T, whereas only lung cancer was linked with 1790G/A. However, the odds ratio values in some of the subgroup analyses were large and lacked statistical power because of the significant heterogeneity. Ethnicity may also significantly affect cancer susceptibility. For the 1790G/A polymorphism, we did not find any association between the 1790G/A polymorphism and cancer risk in Asians. This finding can be explained by the difference in genetic background, environmental exposure, and risk factors relating to lifestyle between Asian and Caucasian populations.

 Some limitations of this meta-analysis should be addressed. First, the lack of the the detailed information about environment risk factors for cancer risk from included studies limited our further evaluation of potential gene–gene and gene–environment interactions. Second, the *P* value of the Hardy–Weinberg equilibrium of three included studies was less than 0.05, suggesting that these study populations were not representative of the broader target population. Despite these limitations, our meta-analysis had some strong advantages. This meta-analysis shed light on the association between HIF-1α polymorphisms and increased risk for various cancers. In addition, the quality of the included studies was satisfactory and met our inclusion criterion. Moreover, substantial numbers of cases and controls were pooled from different studies, which significantly increased the statistical power of the analysis. No publication bias was also found in the collected data.

In summary, this meta-analysis provided insights into the association of HIF-1α 1772 C/T and 1790 G/A gene polymorphisms with cancer risk, supporting the hypothesis that HIF-1α polymorphisms are a susceptibility marker of cancer. However, large sample studies are warranted to validate our findings, especially in some types of cancer, such as breast cancer and cervical cancer. More studies on gene–gene and gene–environment interactions should also be considered in the future to obtain a more comprehensive understanding of the association between HIF-1α polymorphisms and cancer risk.

## Supporting Information

Checklist S1 **PRISMA checklist.**
(DOC)
